# Cytoplasmic maspin expression predicts poor prognosis of patients with soft tissue sarcomas

**DOI:** 10.1186/s13000-014-0205-9

**Published:** 2014-10-30

**Authors:** Chikako Takeda, Yuzo Takagi, Tatsushi Shiomi, Kanae Nosaka, Hideki Yamashita, Mari Osaki, Koji Endo, Takeshi Minamizaki, Ryota Teshima, Hideki Nagashima, Yoshihisa Umekita

**Affiliations:** Division of Organ Pathology, Department of Pathology, Faculty of Medicine, Tottori University, 86 Nishicho, Yonago, Tottori 683-8503 Japan; Division of Orthopedic Surgery, Department of Surgery, Faculty of Medicine, Tottori University, 86 Nishicho, Yonago, Tottori 683-8503 Japan; Department of Orthopedic Surgery, National Hospital Organization Yonago Medical Center, 4-17-1 Kuzumo, Yonago, Tottori 683-0006 Japan

**Keywords:** Maspin, Immunohistochemistry, Soft tissue sarcoma

## Abstract

**Background:**

Maspin is a 42 kDa protein known to act as a tumor suppressor. Although its function has not been fully elucidated, numerous reports have investigated the prognostic impact of maspin in patients with several types of cancer. However, there have been no reports on the association between maspin expression and the prognosis of patients with soft tissue sarcomas (STS). The aim of this study was thus to explore the association of maspin expression with the prognosis of patients with STS.

**Methods:**

One-hundred and eight paraffin-embedded STS tissue samples were immunohistochemically analyzed using antibodies for maspin and Ki-67 antigen. The patients were followed up for 1 to 300 months (median: 33 months) and the prognostic value was evaluated by log-rank test and Cox’s regression hazard model.

**Results:**

Cytoplasmic maspin expression was observed in 48.1% of specimens, and was significantly correlated with a higher FNCLCC grade (*P* = 0.002) and the presence of distant metastases (*P* = 0.001), and those with cytoplasmic maspin expression had both shorter disease-free survival (DFS) and overall survival (OS) by log-rank test (*P* <0.001, *P* = 0.001, respectively). By Cox’s multivariate analysis, the presence of distant metastases was the only prognostic factor for DFS and OS.

**Conclusions:**

This is the first report to reveal an association between maspin expression and the prognosis of patients with STS. Although further studies with a larger series of patients and a longer follow-up period will be needed, cytoplasmic maspin expression could be an indicator of unfavorable prognosis in patients with STS.

**Virtual Slides:**

The virtual slide(s) for this article can be found here: http://www.diagnosticpathology.diagnomx.eu/vs/13000_2014_205

## Background

Soft tissue sarcomas (STS) are relatively rare malignant mesenchymal tumors and constitute less than 1.5% of all cancers, with an annual incidence of about 6 per 100,000 persons [1]. Except for a few types of sarcomas, histological typing does not provide sufficient information for predicting the clinical course of the disease [2]. Therefore, grading and staging systems have been used to predict prognosis and to decide on treatment strategies for adult patients with STS. Several reports have described the relationship between the grading system and prognosis of adult patients with STS, and it has been suggested that the histological grade is the most important prognostic factor [3,4]. In addition, the Ki-67 labeling index has also been reported to be a useful predictor for the prognosis in STS patients [5]. Maspin (mammary serine protease inhibitor) is a 42 kDa protein known to act as a tumor suppressor, and is a member of the serine protease inhibitor (serpin) superfamily. Maspin has been shown to inhibit both tumor growth and metastasis in multiple animal models and cancer cell lines [6]. Although the function of maspin has not been fully elucidated, many reports have described the association between maspin expression and clinicopathological factors in several types of cancer such as breast, prostate, gastric, pancreatic, gallbladder, colorectal, and thyroid cancers, and malignant melanoma [7]. We have also reported an association between maspin expression and clinicopathological factors in several types of cancer such as breast cancer [8-10], colorectal cancer [11], endometrioid endometrial carcinoma [12] and ovarian mucinous borderline tumor [13], and have suggested that cytoplasmic maspin expression may be an indicator of poor prognosis. Although only two reports have described maspin expression in STS [14,15], to our knowledge, no reports have investigated the association between maspin expression and the prognosis of patients with STS. The aim of this study was thus to investigate whether cytoplasmic maspin expression could predict the prognosis of patients with STS.

## Methods

### Tissue specimens

All STS tissue specimens were obtained from the Department of Pathology, Tottori University Hospital, and the affiliated teaching hospitals. We obtained 108 specimens from 108 patients who underwent surgery between October 1981 to March 2012. The specimens consisted of 94 primary tumors, 8 recurrent tumors, and 6 metastatic tumors. Information about adjuvant therapy was obtained for 99 of the patients. Neoadjuvant and/or adjuvant chemotherapy were performed in 22 patients, neoadjuvant and/or adjuvant radiotherapy in 23 patients, and both therapies in 22 patients. We also obtained clinical data such as age, gender, tumor localization, tumor size, presence of distant metastases, methods of treatment, and clinical outcome. The median follow-up period was 33 months (range: 1–300 months). A histological diagnosis was established according to the World Health Organization classification [2]. Histological grades were assigned according to the French Federation of the Cancer Center Sarcoma Group (FNCLCC) system. Written informed consent was obtained and the present study was performed with the approval of the Ethics Committee of the Faculty of Medicine, Tottori University (No. 1558).

### Immunohistochemical procedures

All specimens were fixed in 10% buffered formalin and embedded in paraffin wax. After the sections (4 μm-thick) were deparaffinized and endogenous peroxidase activity was blocked, they were pretreated in citrate buffer (0.01 M, pH 6.0) using a microwave oven (RE-DD6-S; Sharp Corporation, Osaka, Japan) for 20 minutes. After cooling to room temperature, the sections were incubated at 4°C overnight with the following primary antibodies: monoclonal anti-human maspin antibody (clone EAW24; diluted 1:150; Leica Biosystems, Newcastle Ltd., UK), and mouse monoclonal anti-Ki-67 antibody (MIB-1; diluted 1:50; Dako, Glostrup, Denmark). The sections were incubated with biotinylated anti-mouse IgG antibody (BA-2000; diluted 1:150; Vector Laboratories, Burlingame, VT) for 20 minutes, followed by streptavidin biotinylated-HRP conjugate (diluted 1:150; Invitrogen Corporation, Camarillo, CA) for 20 minutes. The sections were then incubated with DAB solution (Liquid DAB + Substrate, Imidazole-HCl buffer, pH 7.5, containing hydrogen peroxide and an anti-microbial agent; Dako, Glostrup, Denmark) for 4 minutes, and finally counterstained with hematoxylin. Normal mammary tissue specimens were used as positive controls for maspin expression.

### Evaluation of immunohistochemical findings

The cells were considered positive cells for maspin expression only when cytoplasmic staining was identified. To count the number of positive cells, a 10 × 10 square grid in the eye-piece was used. The sections were scanned at low and high power magnifications covering all fields. At least three areas having the highest degree of positive cells were selected, and typically 400–500 tumor cells in each field were counted irrespective of immunoreactive status. Thereafter, positive cells were counted and the positive ratio was determined. Tumors with more than 10% positive cells were considered positive for the expression of maspin. We observed Ki-67-positive cell nuclei by a CCD camera in the most distinctly labeled area to evaluate Ki-67 expression. Counts were performed using high-magnification fields with the FLOVEL Image Filing System FlvFs (FLOVEL Inc., Tachikawa, Japan). For measurement of Ki-67 expression, a minimum of 1,000 tumor cells were counted, and the labeling index was determined by calculating the number of positive cells as a percentage of total cells. These evaluations were performed independently by two authors (C.T. and Y.U.) who were blinded to the patient outcome data.

### Statistical analysis

Statistical analysis was performed using the Statistical Package for Social Sciences version 21 (IBM SPSS Statistics; IBM Corporation, New York, NY). The association between maspin expression and clinicopathological parameters was evaluated by non-parametric tests. The Mann–Whitney U test and Chi-square test were used when there were two categorical variables of interest and the Kruskal-Wallis test when there were three variables. Survival curves were calculated using the Kaplan-Meier method and compared by the log-rank test. Multivariate analysis was performed using the Cox proportional hazards regression model and the backward selection method. Hazard ratios were reported with 95% confidence intervals (CI). Values of *P* <0.05 were considered to be statistically significant.

## Results

### Clinicopathological features

Table 1 shows the characteristics of the 108 patients with STS. The majority of patients were adults, with a mean age of 58.3 years (range: 4–93 years). 53 patients were <60 years, and 55 were ≥60 years; 65 patients were male and 43 female. There were 29 tumors <5 cm and 79 tumors were ≥5 cm. There were 88 tumors located in the extremities, 18 in the trunk, and 2 in other areas. Distant metastases were absent in 71 patients and present in the remaining 37 patients. Tumors of FNCLCC grades 1, 2, and 3 were identified in 28, 48, and 32 patients, respectively. The tumors consisted of 39 leiomyosarcomas, 34 liposarcomas, 15 synovial sarcomas, 8 myxofibrosarcomas, 6 undifferentiated/unclassified sarcomas, 5 epithelioid sarcomas and 1 rhabdomyosarcoma. The 8 recurrent tumors consisted of 4 leiomyosarcomas, 2 liposarcomas, and 2 undifferentiated/unclassified sarcomas, and the 6 metastatic tumors consisted of 4 leiomyosarcomas, 1 liposarcoma, and 1 rhabdomyosarcoma.

**Table 1:** **Association between maspin expression and clinicopathological variables in 108 soft tissue sarcomas**

	**Maspin expression**		
**Charactaristics**	**Positive**	**Negative**	***P*** **-value**
	**52 cases**	**56 cases**	
Age			
< 60 years	21	32	0.061
≥ 60 years	31	24	
Gender			
Male	33	32	0.318
Female	19	24	
Tumor size			
< 5 cm	15	14	0.407
≥ 5 cm	37	42	
Tumor localization			
Extremity	44	44	0.097
Trunk	6	12	
Others	2	0	
Distant metastasis			
Absent	26	45	0.001
Present	26	11	
FNCLCC grade			
1	8	20	0.002
2	22	26	
3	22	10	
Ki-67 labelling index			
< 10%	32	43	0.065
≥ 10%	20	13	

### Immunohistochemical findings

**Figure 1:**
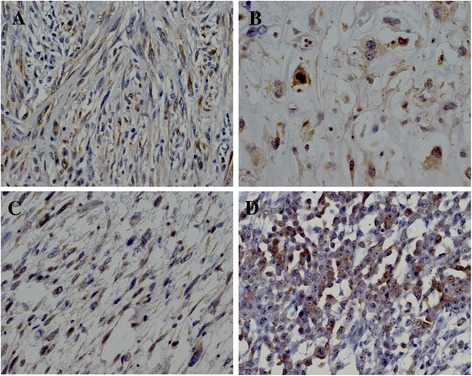
**Representative maspin immunoreactivity in a leiomyosarcoma (A), liposarcoma (B), myxofibrosarcoma (C) and epithelioid sarcoma (D).** (original magnification, ×400).

The mean Ki-67 labeling index of all STS specimens was 8.76 ± 9.77%; 75 cases were labeled <10%, and 33 cases were labeled ≥10% Ki-67 positive. Maspin expression was observed only in the cytoplasm in 52 specimens, in both the cytoplasm and nucleus in 15 specimens, only in the nucleus in 11 specimens, and not at all in 30 specimens; 48.1% of STS specimens were judged to be maspin-positive. By histological type, the number and proportion of maspin positive cases were 22 (56.4%), 8 (23.5%), 11 (73.3%), 2 (25.0%), 4 (66.7%), and 5 (100%) for leiomyosarcoma, liposarcoma, synovial sarcoma, myxofibrosarcoma, undifferentiated/unclassified sarcoma, epithelioid sarcoma, and rhabdomyosarcoma, respectively. The details of maspin expression by histological type are shown in Table 2. Representative images of maspin staining are shown in Figure 1 for a leiomyosarcoma, liposarcoma, myxofibrosarcoma, and epithelioid sarcoma.

**Table 2:** **Maspin expression in 108 soft tissue sarcomas**

**Histologic type**	**Maspin expression**	
	**Positive (%)**	**Negative**
	**52 (48.1)**	**56**
Leiomyosarcoma	22 (56.4)	17
Liposarcoma	8 (23.5)	26
Well-differentiated	5 (29.4)	12
Myxoid	2 (14.3)	12
Pleomorphic	1 (50)	1
Dedifferentiated	0	1
Synovial sarcoma	11 (73.3)	4
Spindle cell	6 (85.7)	1
Biphasic	5 (62.5)	3
Myxofibrosarcoma	2 (25)	6
Undifferentiated/ unclassified sarcoma	4 (66.7)	2
Epithelioid sarcoma	5 (100)	0
Rhabdomyosarcoma	0	1
Embryonal	0	1

### Correlation between maspin expression and clinicopathological factors

Cytoplasmic maspin expression was significantly correlated with distant metastases (*P* = 0.001) and FNCLCC grade (*P* = 0.002). With respect to the FNCLCC grades, a significant correlation was observed between grade 1 and grade 3 (*P* <0.01). Other clinicopathological factor, including age, gender, tumor location and size, and Ki-67 labeling showed no significant correlation with cytoplasmic maspin expression.

### Survival analysis

**Figure 2:**
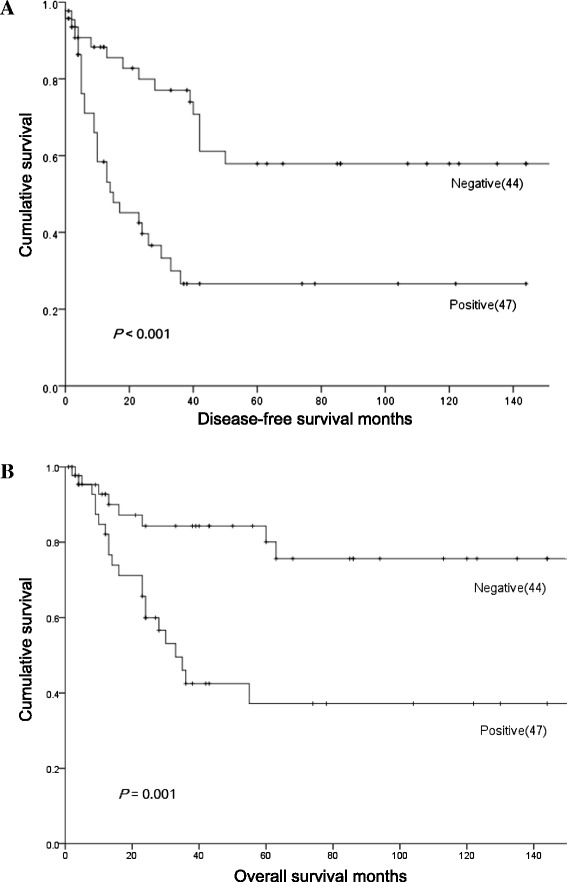
**Disease-free survival (A) and overall survival (B) curves of 91 patients with soft tissue sarcomas according to maspin expression status.**

After the follow-up period, 29 patients had died of STS and 7 patients had died of other causes. Of the remaining 72 patients, 12 patients experienced recurrence of STS. We excluded 17 patients with well-differentiated liposarcomas from the survival analysis due to their being classified as having intermediate, not malignant, tumors; of these patients, 15 were still alive and two had died of other causes. Maspin expression in only cytoplasm was observed in 47 specimens, both in cytoplasm and nucleus in 12 specimens, in only nucleus in 8 specimens, and not at all in 24 specimens. Of the 91 patients analyzed by log-rank test, the patients who were only cytoplasmic expression of maspin showed shorter overall survival (OS) and disease-free survival (DFS) (*P* = 0.001, *P* <0.001, respectively; Figure 2). Univariate analysis showed a significant difference in both DFS and OS according to FNCLCC grade (*P* = 0.015, *P* = 0.016), distant metastases (*P* <0.001, *P* <0.001), Ki-67 labeling index (*P* = 0.016, *P* = 0.031), and the presence of maspin expression (*P* = 0.013, *P* =0.016), respectively. By Cox multivariate analysis, the presence of distant metastases was the only independent prognostic indicator of both DFS and OS (*P* <0.001, *P* <0.001, respectively; Table 3).

**Table 3:** **Multivariate analysis of clinicopathological factors for disease-free survival and overall survival in 91 soft tissue sarcomas**

**Variables**	**Disease-free survival**				**Overall survival**	
	**HR**	**95% CI**	***P*** **-value**	**HR**	**95% CI**	***P*** **-value**
FNCLCC grade						
III vs I,II	1.05	0.533-2.066	0.891	0.82	0.387-1.751	0.614
Distant metastasis						
Present vs absent	12.7	5.682-27.78	< 0.001	55.6	7.575-500	< 0.001
Ki-67 labelling index						
≥10% vs <10%	1.30	0.700-2.392	0.410	1.57	0.754-3.286	0.227
Maspin expression						
Positive vs negative	1.53	0.777-3.005	0.219	1.64	0.708-3.791	0.248

HR, Hazard ratio; 95% CI, 95% confidence intervals.

## Discussion

Maspin is a member of the serpin family of protease inhibitors and was originally thought to be a tumor suppressor due to its ability to inhibit invasion, motility, and metastasis of mammary tumors [16]. However, loss of maspin expression in several cancers, such as pancreatic, colorectal and ovarian cancer, is not commonly observed due to a lack of maspin expression in the corresponding normal tissue. The most compelling data regarding the clinical significance of maspin in cancer progression and metastasis emerged from survival studies of cancer patients. Although the original studies revealed an association between reduced maspin expression and cancer progression and worse prognosis, it has been demonstrated that this correlation was far more complex than originally suspected. Factors contributing to this complexity include the differences in cancer type (e.g. adenocarcinoma vs squamous cell carcinoma), cut-off values of positive criteria, antibodies used, methods of detection, and subcellular maspin distribution. The subcellular localization of maspin is predominantly cytoplasmic; however, maspin exerts its effect in the nucleus at the level of gene and chromatin regulation, and is released only as a consequence of cell damage or necrosis [17,18]. Goulet *et al*. demonstrated that nuclear localization of maspin was essential for its inhibition of tumor growth and metastasis [19]. Sood *et al*. reported that nuclear maspin staining was associated with increased survival, whereas cytoplasmic maspin staining was associated with a poor outcome in ovarian carcinoma [20]. Additionally, Marioni *et al*. reported that nuclear maspin expression was associated with a lower recurrence rate and a longer disease-free interval after surgery for squamous cell carcinoma of the larynx [21]. We have also reported that cytoplasmic maspin expression was associated with an aggressive phenotype and poor prognosis of patients with breast cancer [8-10], colorectal cancer [11], and endometrioid endometrial carcinoma [12]. On the other hand, there have been few reports investigating maspin expression in non-epithelial tissue [22], and only two reports have described the expression of maspin in STS. Kim *et al*. reported a case of metastatic leiomyosarcoma from a uterus showing expression of maspin in addition to several types of growth factors, angiogenic factors, and proliferative markers in the metastatic tumor cells by immunohistochemistry and immunoblot detection [14]. Although they did not describe the subcellular localization of maspin expression, they revealed that the maspin protein was more intensely expressed in the metastatic tumor compared to the primary uterine leiomyosarcoma. In the present study, we observed the expression of maspin in 56.4% of leiomyosarcomas. These results highlight the need for further studies on the use of maspin expression as a prognostic indicator in leiomyosarcomas. Fitzgerald *et al*. reported that chondrosarcoma cells exhibited upregulated maspin mRNA expression in addition to decreased DNA methylation of the maspin gene [15]. They also demonstrated that the upregulation of maspin mRNA may either play an important role in malignant progression, or simply be a biomarker of tumor progression. Their findings may support our present results that expression of maspin is correlated with the poor prognosis of patients with STS, although chondrosarcomas were not investigated in our study.

## Conclusion

In conclusion, to our knowledge, this is the first report investigating the association of maspin expression with the prognosis of patients with STS. Although further studies with a larger series of patients and a longer follow-up period should be requisite, the present study suggests the potential usefulness of cytoplasmic maspin expression as a prognostic factor of patients with STS. Further carefully designed studies will be needed to elucidate the function and role of cytoplasmic maspin expression in patients with STS.
